# Ranking Transitive Chemical-Disease Inferences Using Local Network Topology in the Comparative Toxicogenomics Database

**DOI:** 10.1371/journal.pone.0046524

**Published:** 2012-11-07

**Authors:** Benjamin L. King, Allan Peter Davis, Michael C. Rosenstein, Thomas C. Wiegers, Carolyn J. Mattingly

**Affiliations:** 1 Mount Desert Island Biological Laboratory, Salisbury Cove, Maine, United States of America; 2 North Carolina State University, Raleigh, North Carolina, United States of America; Laurentian University, Canada

## Abstract

Exposure to chemicals in the environment is believed to play a critical role in the etiology of many human diseases. To enhance understanding about environmental effects on human health, the Comparative Toxicogenomics Database (CTD; http://ctdbase.org) provides unique curated data that enable development of novel hypotheses about the relationships between chemicals and diseases. CTD biocurators read the literature and curate direct relationships between chemicals-genes, genes-diseases, and chemicals-diseases. These direct relationships are then computationally integrated to create additional inferred relationships; for example, a direct chemical-gene statement can be combined with a direct gene-disease statement to generate a chemical-disease inference (inferred via the shared gene). In CTD, the number of inferences has increased exponentially as the number of direct chemical, gene and disease interactions has grown. To help users navigate and prioritize these inferences for hypothesis development, we implemented a statistic to score and rank them based on the topology of the local network consisting of the chemical, disease and each of the genes used to make an inference. In this network, chemicals, diseases and genes are nodes connected by edges representing the curated interactions. Like other biological networks, node connectivity is an important consideration when evaluating the CTD network, as the connectivity of nodes follows the power-law distribution. Topological methods reduce the influence of highly connected nodes that are present in biological networks. We evaluated published methods that used local network topology to determine the reliability of protein–protein interactions derived from high-throughput assays. We developed a new metric that combines and weights two of these methods and uniquely takes into account the number of common neighbors *and* the connectivity of each entity involved. We present several CTD inferences as case studies to demonstrate the value of this metric and the biological relevance of the inferences.

## Introduction

**Figure 1:**
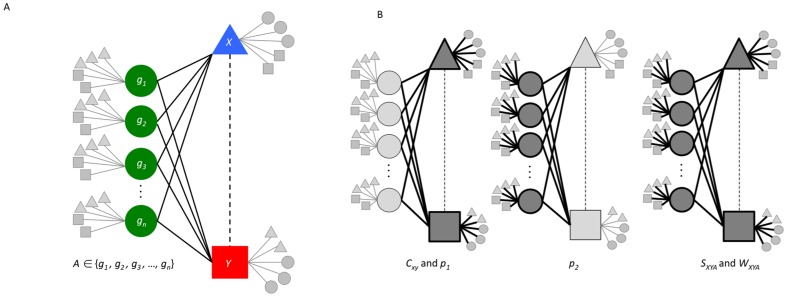
Transitive chemical-disease inferences and the computational approaches used to score inferences. A) Diagram of local network for the transitive chemical-disease inference (dotted line) between a chemical, *X*, and a disease, *Y*, using a set of genes, *A*, that have both curated chemical-gene interactions and gene-disease associations (solid lines). The chemical, disease and each gene involved have interactions and relationships to other nodes (chemicals, genes, diseases) in the database. Chemical *X* has some number of other genes (grey circles) that it interacts with and associated diseases (grey squares). Disease *Y* has other associated genes and curated relationships to other chemicals (grey triangles). Each gene used to make the inference, *g_1_* to *g_n_*, are known to interact with other chemicals (grey triangles) and are associated with other diseases (grey squares). B) Diagrams showing three methods to score inferences. The first, *C_XY_* and *p_1_*, is based on the number of genes (circles) used to make the inference and the connectivity (bold lines) of the chemical (triangle) and disease (square). The second, *p_2_*, takes the number of genes (circles) used to make the inference and their connectivity (bold lines) into account. The third, *S_XYA_* and *W_XYA_*, takes the number of genes into account as well as the connectivity of the chemical, disease and each of the genes into account.

A consequence of our highly industrialized society is exposure to an increasing number of chemicals that may influence human health. Environmental factors are implicated in many complex diseases including asthma, cancer, diabetes and Parkinson's disease. However, the mechanisms of actions of most chemicals and the etiologies of environmentally influenced diseases are not well understood [1]. The Comparative Toxicogenomics Database (CTD; http://ctdbase.org) promotes understanding about the effects of environmental chemicals on human health [2]. CTD integrates manually curated data reported in the peer-reviewed literature with select public data sets to provide a freely available resource for exploring cross-species chemical-gene and protein interactions and chemical- and gene-disease relationships. CTD provides transitive inferences between chemicals, genes and diseases that are intended to help users develop experimentally testable hypotheses about mechanisms of chemical actions and disease etiologies. A transitive inference between a chemical and disease is made when one or more genes have curated interactions with the chemical and the disease (Figure 1A). Likewise, a transitive inference between a gene and disease is made when one or more chemicals have curated interactions with the gene and the disease. In CTD, there are two classes of transitive inferences: a) inferred relationships that also have direct evidence curated from the published literature and b) inferred relationships that do not yet have directly curated evidence. Recent reports citing Swanson's ABC model underscore the potential value of transitive inferences for predicting disease treatments [3], [4], [5]. Data in CTD facilitate similar discovery processes for chemical-gene-disease interaction networks.

All inferences in CTD are built upon manually curated chemical-gene interactions, gene-disease relationships or chemical-disease (C–D) relationships. Integration of these components allows inferences to be constructed reciprocally. For example, inferred chemical relationships can be viewed for a given disease and inferred disease relationships can be viewed for a given chemical. The former provide insights into the potential environmental influences on a disease, whereas the latter provide insight into the potential health effects of exposure to a chemical. The gene sets that underlie these inferences are unique to CTD and provide a foundation for developing novel hypotheses about the mechanisms by which specific environmental factors affect human health. (Analogous data are provided for gene-disease inferences). As the data in CTD have grown, the number of inferences has increased exponentially. To assist users with interpretation and prioritization of inferences, we developed a statistical method for ranking CTD inferences.

We modeled CTD data as a network where chemicals, genes and diseases are nodes, and the relationships between them are edges. Like other biological networks, the CTD network is a scale-free random network that contains highly connected hub nodes [6]. The presence of hubs introduces a statistical challenge when evaluating networks, as not all edges are equally likely to occur. For C–D inferences, we construct a local network that consists of the chemical, disease and the set of genes that interact with the chemical and the disease. To rank order C–D inferences, the similarity among the local networks have to be compared. In these comparisons, hub nodes will appear in multiple local networks by chance and make inferences appear more similar unless they are discounted. The following example illustrates the scale of this statistical problem both in terms of the number of disease inferences for a chemical and the topology of the local network for a particular C–D inference.

Bisphenol A (BPA) is a ubiquitous endocrine disruptor that has been associated with developmental abnormalities and cancer [7], [8]. In the July 2011 release of CTD, BPA had abundant and varied types of C–D relationships including four that were directly curated, seven that were curated and inferred, and 798 that were only inferred. BPA was associated with breast neoplasms based on both curated evidence [9] as well as by inference via 73 common interacting genes. The local network for this inference consists of the chemical (BPA), the disease (breast neoplasms) and each of the 73 genes. A subset of these 73 genes is also associated with many other diseases and chemicals. In this example, such hub genes include *BCL2, CYP1A1, ESR1, IL1B, NOS2, PTGS2, TNF* and *TP53*, each of which have over 400 curated interacting chemicals. In addition, BPA and breast neoplasms have been targeted for in-depth CTD curation and are hubs themselves. BPA has curated interactions with 1,235 genes, and breast neoplasms has 266 curated gene relationships. In developing a mechanism for statistically ranking inferences, it was also important to determine the relative influence of hub versus non-hub data.

Two previously published studies used local topology-based statistics to assess the reliability of protein-protein interactions generated from high-throughput assays, such as yeast two-hybrid technology [10], [11]. These studies examined the reliability of an interaction between two proteins (A and B) based on how many *other* proteins (called common neighbors) interacted with A and B. These data were modeled as a network where each protein was a node and the interactions were edges connecting the nodes. The number of interactions for a node are defined as the node degree. Goldberg and Roth [12] applied four different methods to calculate a probability that a given interaction between proteins A and B was reliable based on the node degree of A and B and the number of additional proteins that interacted with both A and B. Among these methods, the hypergeometric clustering coefficient performed best, but this method did not take into account the node degree of the additional proteins. Li and Liang [13] developed two common neighbor statistics to assess the reliability of a given protein-protein interaction. Similar to the hypergeometric clustering coefficient, one metric (*p_1_*) took into account the number of common neighbors and the degree of the two proteins that form the interaction of interest. The second metric (*p_2_*) took into account the degree of each common neighbor. The authors presented a sequential process of evaluating interactions with each statistic rather than presenting a combined statistic. We explored whether these methods could be modified for ranking C–D inferences by substituting protein A with a chemical, protein B with a disease, and the common protein neighbors with the set of genes underlying a C–D inference.

Here, we present a novel method that *combines and weights* the *p_1_* and *p_2_* metrics, taking into account the properties of the local networks containing the chemical, disease and each of genes used to make CTD inferences. This method addresses the challenges presented by the large number of possible inferences, as well as the presence of hub data. The score rewards inferences by the number of genes used to make the inference, and penalizes networks containing nodes where the node degree is high. Figure 1B illustrates the difference between the hypergeometric clustering coefficient and the *p_1_* and *p_2_* metrics. We provide several examples to demonstrate the value of the statistic as well as the biological relevance of the inferences.

## Results

### Transitive Chemical-Disease Inferences in CTD

We modeled the associations among chemicals, genes and diseases in CTD as a binary tripartite network. The network is tripartite because it comprises three types of nodes: chemicals, genes and diseases. Associations between the nodes were modeled as binary edges that had a value of either present or absent. As the node degree influences the number of transitive inferences that can be made, we investigated the distribution of degrees for all nodes. Like other biological networks, the CTD network was found to be a scale-free random network where node degree can be described by the power-law distribution (Figure S1). The observed distribution shows that the degree of nodes was not uniform. Instead, 89% of nodes have fewer than 20 edges and there are just a few hub nodes. The connectivity of chemicals, genes and diseases in CTD reflects one or more factors including a) biological function, b) representation in the peer-reviewed scientific literature, or c) the current level of manual curation for an entity. Table 1 shows the top 20 hub chemicals, genes and diseases in the July 2011 release of CTD. The top ten chemicals and diseases reflect the large volume of published studies and priority areas for CTD curation. The high connectivity of the top ten genes reflects their roles in diverse curated chemical and disease processes. Indeed, *TNF* is involved in 46,587 C–D inferences alone. Any analyses done on scale-free random networks must take network topology into account due to the presence of hub nodes. Accounting for topology is especially important when examining inferences, as they could be solely based on hub nodes rather than a combination of hub and low-degree nodes. The purpose of using a topologically based method was to reduce the influence of hub nodes in C–D inferences.

**Table 1:** Top 20 hub chemicals, genes and disease in the CTD network.

Chemical Name (ID)	Edges	Gene Symbol	Edges	Disease Name (ID)	Edges
Tetrachlorodibenzodioxin (D013749)	7176	*TNF*	835	Prostatic Neoplasms (D011471)	515
Acetaminophen (D000082)	6362	*CASP3*	581	Breast Neoplasms (D001943)	442
pirinixic acid (C006253)	5664	*CYP1A1*	553	Autistic Disorder (D001321)	303
Ammonium Chloride (D000643)	5271	*MAPK1*	551	Lung Neoplasms (D008175)	240
Ethinyl Estradiol (D004997)	5066	*MAPK3*	546	Liver Cirrhosis, Experimental (D008106)	230
Cyclosporine (D016572)	4601	*PTGS2*	521	Stomach Neoplasms (D013274)	210
Benzo(a)pyrene (D001564)	3397	*IL6*	517	Colorectal Neoplasms (D015179)	197
7,8-Dihydro-7,8-dihydroxybenzo(a)pyrene 9,10-oxide (D015123)	2918	*IL1B*	492	Craniofacial Abnormalities (D019465)	179
4,4′-diaminodiphenylmethane (C009505)	2702	*CYP1A2*	473	Carcinoma, Hepatocellular (D006528)	173
2,4-dinitrotoluene (C016403	2647	*TP53*	458	Drug-Induced Liver Injury (D056486)	167
2,6-dinitrotoluene (C023514)	2628	*NOS2*	456	Melanoma (D008545)	157
Estradiol (D004958)	2620	*ESR1*	453	Colonic Neoplasms (D003110)	126
Tamoxifen (D013629)	2259	*BCL2*	443	Inflammation (D007249)	124
Carbon Tetrachloride (D002251)	2237	*CYP3A4*	414	Liver Diseases (D008107)	122
Diethylnitrosamine (D004052)	2153	*FOS*	404	Liver Neoplasms (D008113)	122
Tretinoin (D014212)	1957	*BAX*	380	Neoplasms (D009369)	118
arsenic trioxide (C006632)	1938	*CDKN1A*	375	Schizophrenia (D012559)	115
sodium arsenite (C017947)	1910	*HMOX1*	374	Alzheimer Disease (D000544)	109
Dietary Fats (D004041)	1907	*RELA*	368	Leukemia, Myeloid, Acute (D015470)	107
Phenobarbital (D010634)	1831	*IL8*	367	Adenocarcinoma (D000230) and Seizures (D012640)	95

Transitive C–D inferences are made in CTD when a chemical is known to interact with one or more genes that are also associated with a disease. In the July 2011 data release, CTD contained curated data from 26,247 references for 6,406 chemicals, 20,898 genes and 3,999 diseases. Using these data, a total of 338,484 C–D transitive inferences were made for 5,959 chemicals and 3,305 diseases. Because of hub chemicals, genes and diseases, and the underlying scale-free properties of the CTD network, the proportion of disease inferences per chemical is not uniform. For example, warfarin has curated interactions with just 32 genes that are used to make 164 C–D inferences. Warfarin has 55 edges making it a relatively high-degree chemical as 92% of chemicals have fewer edges. In contrast, BPA is a hub chemical with a total of 1,247 edges. BPA has 1,235 gene interactions and 858 C–D inferences. All but eight of BPA's inferences appear novel because either direct evidence in the literature is lacking or the evidence has not yet been curated for CTD. While novel inferences may generate new hypotheses about environmental influences on diseases, in the absence of a ranking metric, the large number of inferences makes it challenging to prioritize them for further investigation.

### Scoring Chemical-Disease Inferences

To facilitate interpretation and prioritization of inferences for hypothesis development and further study, we explored statistical methods that would allow C–D inferences to be ranked. We compared results from several methods developed to study the reliability of protein-protein interactions. All C–D inferences in CTD were analyzed using: the hypergeometric clustering coefficient (*C_xy_*) [12]; the two common neighbor statistics (*p_1_* and *p_2_*) [13]; and two novel variants on these metrics, including the product (*S_XYA_*) and weighted product (*W_XYA_*) of those statistics. We evaluated these four metrics by comparing:

1. The ranked order of disease inferences for a given chemical in different contexts.
2. C–D inferences with particular local network topological features versus curated C–D relationships.
3. The extent to which the *C–D curated relationships* supported the relative rankings of *C–D inferences versus following data randomization*.

#### Ranked order of disease inferences

Due to scale-free random network properties of the CTD network, many chemicals have a large number of disease inferences. Initially, the C–D inferences were ranked first by the presence of curated evidence and then by the number of common interacting genes. Although the number of interacting genes was useful for conveying the current state of the data, this metric alone failed to take into account the context of these genes. For example, many “ties” existed where disease inferences were based on the same number of genes, regardless of the differences among the genes. Table 2 provides 21 disease inferences for BPA that are based on sets of five genes. Among these inferences, 20 involve at least one gene with more than 100 edges. We applied *C_xy_*, *p_1_*, *S_XYA_* and *W_XYA_* statistics to determine whether their inclusion of contextual information would distinguish between these “ties.” Table 2 shows how the five different statistics for these 21 BPA-disease inferences can start to rank and order the inferences (e.g., Disorders of Sex Development vs. Kidney Diseases), even though all 21 inferences are made via five genes each. Consistently, we found that *S_XYA_* and *W_XYA_* had the lowest frequency of ties among all inferences (Figure S2). Consistent with this observation, we also found that inferences based on fewer rather than larger genes were often scored higher using these two metrics. For example, the inference between BPA and Female Urogenital Diseases involved seven genes (*ESR1, HOXA10, HOXA11, IGF1, LIF, WNT4, WNT5A*) and had a *W_XYA_* score of 12.93, which was higher than an inferred relationship with Rheumatoid Arthritis that involved nine genes (*AHR, ENO1, IL18, LCN2, MMP2, PTGS2, PTPRC, TNF, VEGFA*) and had a score of 6.96. The more significant score for Female Urogenital Diseases reflected the lower connectivity of the genes involved in this inference. For these two inferences, the geometric mean of the node degree was 55.9 for Female Urogenital Diseases versus 160.7 for the Rheumatoid Arthritis.

**Table 2:** Disease inferences for BPA that are based on five interacting genes.

Disease (# edges)	Gene Symbols (# edges)	*C_xy_*	*p_1_*	*p_2_*	*S_XYA_*	*W_XYA_*
Disorders of Sex Development (MESH:D012734) (5)	*CYP19A1* (202); *HSD17B3* (27); *LHB* (83); *LHCGR* (34); *NR3C1* (189)	7.48	17.21	27.02	22.04	17.10
Muscular Dystrophy, Facioscapulohumeral (MESH:D020391) (9)	*CDKN1A* (375); *DCN* (49); *ELN* (32); *HSPA1B* (84); *LUM* (25)	5.42	12.51	27.77	20.21	12.45
Osteosarcoma (MESH:D012516) (11)	*CYP3A4* (414); *FOLR1* (39); *JUN* (305); *NR1I2* (216); *TP53* (458)	4.88	11.27	22.54	16.91	11.38
Metabolic Syndrome X (MESH:D024821) (16)	*ADIPOQ* (89); *CCL2* (283); *LEP* (106); *SHBG* (48); *TRIB3* (51)	3.96	9.19	26.30	18.36	10.27
Precancerous Conditions (MESH:D011230) (20)	*CCND1* (327); *IRS1* (47); *MAPK8* (200); *MAPK9* (109); *PTGS2* (521)	3.46	8.05	23.44	15.74	8.19
Myocardial Reperfusion Injury (MESH:D015428) (22)	*ADIPOG* (89); *EDN1* (186); *NOS2* (456); *PTEN* (100); *SLC8A1* (31)	3.25	7.59	25.19	16.39	7.75
Drug Hypersensitivity (MESH:D004342) (22)	*ABCC2* (204); *HSPA1A* (122); *IL4* (228); *IL4RA* (17); *TNF* (835)	3.25	7.59	24.13	15.86	7.74
Limb Deformities, Congenital (MESH:D017880) (25)	*CACNA1C* (23); *FGFR2* (90); *HOXA11* (22); *TBX3* (21); *TGFB2* (70)	2.98	6.98	30.32	18.98	7.41
Carcinoma (MESH:D002277) (25)	*BCL2* (443); *EGFR* (204); *KRAS* (79); *PTGS2* (521); *TARBP2* (11)	2.98	6.98	24.73	15.86	7.14
Endometrial Neoplasms (MESH:D016889) (34)	*BIRC5* (167); *DCN* (49); *HOXA11* (22); *PTEN* (100); *SUZ12* (13)	2.37	5.62	29.24	17.79	6.07
Cleft Lip (MESH:D002971) (28)	*FGFR1* (73); *FGFR2* (90); *FGFR3* (60); *SPRY2* (19); *TYMS* (113)	2.75	6.46	28.09	17.28	5.83
Leukemia, Promyelocytic, Acute (MESH:D015473) (34)	*AKT1* (315); *CD44* (70); *CEBPA* (78); *ITGB2* (46); *RARA* (81)	2.37	5.62	26.32	15.97	5.81
Dermatitis, Atopic (MESH:D003876) (37)	*CXCL10* (87); *IFNG* (347); *IL1B* (492); *IL4* (228); *TSLP* (18)	2.21	5.27	24.37	14.82	5.44
Neuroblastoma (MESH:D009447) (39)	*IFNB1* (48); *MET* (61); *MYC* (253); *MYCN* (31); *NTRK2* (37)	2.11	5.05	28.09	16.57	5.26
Glioblastoma (MESH:D005909) (42)	*IL1B* (492); *MMP2* (194); *NCOR1* (39); *TGM2* (75); *VEGFA* (271)	1.97	4.76	24.17	14.46	4.94
Cardiovascular Diseases (MESH:D002318) (43)	*CBS* (24); *CCL2* (283); *EDN1* (186); *GH1* (70); *VCAM1* (123)	1.93	4.67	25.86	15.27	4.86
Liver Cirrhosis (MESH:D00103) (49)	*CTGF* (77); *FGFR2* (90); *MMP2* (194); *SPP1* (113); *THBS1* (68)	1.70	4.18	25.90	15.04	4.38
Colitis, Ulcerative (MESH:D003093) (50)	*GNA12* (18); *IL12B* (194); *IL1B* (492); *PTPN2* (18); *STAT3* (127)	1.67	4.11	26.77	15.44	4.32
Cell Transformation, Neoplastic (MESH:D002471) (60)	*NOS2* (456); *SLC16A1* (73); *SLC2A1* (88); *TSC22D1* (46); *WNT5A* (38)	1.37	3.48	26.52	15.00	3.70
Lupus Erythematosus, Systemic (MESH:D008180) (62)	*CLU* (115); *ETS1* (35); *FASLG* (72); *IL12B* (194); *IL4* (228)	1.32	3.38	25.71	14.55	3.58
Kidney Diseases (MESH:D007674) (71)	*CDKN1A* (375); *HOXA11* (22); *LCN2* (64); *LRP2* (19); *TERT* (80)	1.11	2.97	28.15	15.56	3.20

**Figure 2:**
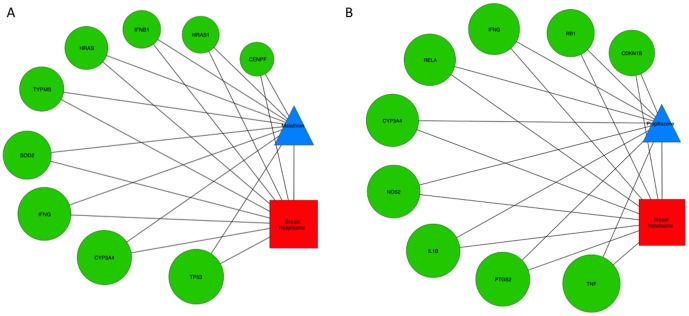
Example chemical-disease inference networks with similar numbers of genes, but with different node degrees. **A**) Malathion-Breast Neoplasms inference network with *C_xy_* = 7.49, *p_1_* = 17.30, *p_2_* = 40.32, *S_XYA_* = 28.81 and *W_XYA_* = 17.31 that used the following genes (degrees listed in parentheses and used to set gene node diameter): CENPF (29), CYP3A4 (414), HRAS (95), HRAS1(42) IFNB1 (48), IFNG (347), SOD2 (191), TP53 (458) and TYMS (113). **B**) Pioglitazone-Breast Neoplasms inference network with *C_xy_* = 7.24, *p_1_* = 16.72, *p_2_* = 32.10, *S_XYA_* = 24.46 and *W_XYA_* = 16.73 that used the following genes: CDKN1B (167), CYP3A4 (414), IFNG (347), IL1B (492), NOS2 (456), PTGS2 (521), RB1 (209), RELA (368) and TNF (835).

Four of the five statistics take the degree of the chemical and disease and the number of genes used to make the inference into account. We compared these four statistical methods (*C_xy_*, *p_1_*, *S_XYA_* and *W_XYA_*) by examining C–D inferences for which the degrees of the *chemical and disease were similar and the number of genes involved were the same*. We provide an example involving the chemicals, malathion and pioglitazone, and their inferred relationships to Breast Neoplasms (Figure 2). Although both relationships have supporting evidence in the literature, CTD also infers these relationships based on common interacting genes. Malathion is an organophosphorous pesticide that has been shown to induce malignant transformation in a human breast epithelial cell line [14]. Pioglitazone is an activator of peroxisome proliferator-activated receptor gamma that has been used to treat Type 2 Diabetes, and in this context, was correlated with a decreased incidence of breast cancer [15]. In the local network for each of these inferences, malathion has 54 edges, pioglitazone has 60 edges and the disease has 443 edges. In both cases, each inference is based on nine genes; however, the gene sets differ in content. Both include *CYP3A4* and *IFNG*, but the degrees for the seven remaining genes underlying the pioglitazone inference are much higher than those for the malathion inference. Each of the pioglitazone inference genes has at least 167 edges with a geometric mean of 383.6 edges for the full gene set, whereas the malathion inference has five genes with fewer than 167 edges and a geometric mean of 125.8 edges for the full gene set. *C_xy_* and *p_1_* statistics could not distinguish between these inferences as they do not consider the degree of the genes. In contrast to the four statistics (*C_xy_*, *p_1_*, *S_XYA_* and *W_XYA_*), the *p_2_* statistic calculates the degree of the genes, and consequently, the malathion inference score is higher since the degrees of five of its underlying genes (*CENPF, HRAS, HRAS1, IFNB1 and TYMS)* are lower than the least connected gene (*CDKN1B)* for the pioglitazone inference. In this example, the aggregate statistic, *S_XYA_*, and the weighted aggregate, *W_XYA_*, both ranked the Breast Neoplasms inference as more significant for malathion than for pioglitazone since it includes *p_2_*.

#### Local network topological features versus curated C–D relationships

Both aggregate statistics (*S_XYA_* and *W_XYA_*) consider the degrees of the chemical, disease and genes in addition to the number of genes involved (*m*) and, thus, offer advantages over the two statistics that do not (*C_xy_*, *p_1_*). The unweighted aggregate statistic, *S_XYA_*, was our first attempt to combine *p_1_* and *p_2_*, but we found it to be highly correlated with *m*. We explored several ways to weight the calculation using constant values or values proportional to *m*. To objectively evaluate these weighting schemes, we leveraged the curated C–D relationships in CTD to determine whether the ranking of inferences from a given method would correlate with whether the inferences *also* had curated evidence (i.e., would inferences that are additionally supported by curated evidence rank higher than those without). It is important to note that in the examples studied, curated evidence was derived from sources independent of those involved in forming the inference. We evaluated the top 100 scoring inferences (Figure S3) and found that *W_XYA_* ranked the C–D inferences with curated evidence higher than S*_XYA_*. In fact, six of the top ten scoring inferences ranked by *W_XYA_* are C–D inferences that also have curated evidence (Table 3A) whereas only three of the top ten scoring inferences ranked by *S_XYA_* were validated by curated data (Table 3B). The six curated inferences ranked highest by *W_XYA_* included a range of diseases and chemicals (Table 3A). In contrast, all but one of top ten inferences ranked highest by *S_XYA_* involved prostatic neoplasms (Table 3B). Prostatic neoplasms is the top disease hub node in CTD and dominates the top inferences by *S_XYA_* since *p_1_* is large with so many genes involved in those inferences. Because *W_XYA_* weights *p_1_* by the number of genes involved, prostatic neoplasms does not dominate the list of top ranked inferences. Upon further examination of the published literature, all “novel” inferences among the top ten ranked inferences by *W_XYA_* are substantiated. Ammonium chloride induced hypertrophy in the DU-145 prostatic cell line [16]. A pilot study of Vietnam veterans exposed to tetrachlorodibenzodioxin had increased risk for developing prostate cancer [17]. Rats exposed to nitrobenzenes were reported to have lesions in several organs including the liver [18]. Vorinostat is currently being evaluated as a therapeutic for acute myeloid leukemia [19]. In a similar fashion, all but two (pirinixic acid and biethylnitrosamine and prostatic neoplasms) of the top ten inferences are substantiated by further examination of published literature. Together, these results demonstrate that ranking inferences using *W_XYA_* is less biased by major hub nodes than *S_XYA_*, and *W_XYA_* may, therefore, be a potentially valuable predictor of novel C–D relationships.

**Table 3:** Top ten C–D inferences by *W_XYA_* (a). and *S_XYA_* (b).

(a)					
Chemical	Disease	*m*	*S_XYA_*	*W_XYA_*	Comment
decitabine (C014347)	Stomach Neoplasms (D013274)	102	435.03	241.34	Curated [35]
Dimethylnitrosamine (D004128)	Liver Cirrhosis, Experimental (D008106)	80	343.09	224.11	Curated (several references in CTD)
Estradiol (D004958)	Prostatic Neoplasms (D011471)	186	628.38	196.16	Curated [36]
Arsenic (D001151)	Arsenic Poisoning (D020261)	55	271.92	192.09	Curated [37]
nitrofen (C007350)	Hernia, Diaphragmatic (D006548)	35	194.42	189.09	Curated (several references in CTD)
Ammonium Chloride (D000643)	Prostatic Neoplasms (D011471)	242	795.14	174.28	Novel inference
Tetrachlorodibenzodioxin (D013749)	Prostatic Neoplasms (D011471)	280	921.97	172.74	Novel inference
Arsenic (D001151)	Skin Diseases (D012871)	55	259.37	171.16	Curated [37]
Nitrobenzenes (D009578)	Liver Diseases (D008107)	29	175.48	170.82	Novel inference
vorinostat (C111237)	Leukemia, Myeloid, Acute (D015470)	38	201.93	164.18	Novel inference

(b)					
Chemical	Disease	*m*	*S_XYA_*	*W_XYA_*	Comment
Tetrachlorodibenzodioxin (D013749)	Prostatic Neoplasms (D011471)	280	921.97	172.74	Novel inference
Ammonium Chloride (D000643)	Prostatic Neoplasms (D011471)	242	795.14	174.28	Novel inference
Ethinyl Estradiol (D004997)	Prostatic Neoplasms (D011471)	216	711.75	137.84	Novel inference
pirinixic acid (C006253)	Prostatic Neoplasms (D011471)	211	680.16	111,31	Novel inference
Acetaminophen (D000082)	Prostatic Neoplasms (D011471)	211	670.44	91.54	Novel inference
Estradiol (D004958)	Prostatic Neoplasms (D011471)	186	628.38	196.16	Curated [36]
Cyclosporine (D016572)	Prostatic Neoplasms (D011471)	172	548.78	87.27	Novel inference
Tetrachlorodibenzodioxin (D013749)	Breast Neoplasms (D001943)	177	538.91	59.82	Curated [38]
Benzo(a)pyrene (D001564)	Prostatic Neoplasms (D011471)	152	481.94	96.56	Curated [39]
Diethylnitrosamine (D004052)	Prostatic Neoplasms (D011471)	142	466.03	135.53	Novel inference

#### Corroboration of C–D curated relationships of actual vs. randomized inferences

The extent to which specific chemicals give rise to disease is not well understood. Consequently, there is no strong validation set with which to estimate the rate of true positives vs. false positives. As a possible alternative, we explored two approaches that utilized CTD data to assess the validity of particular inferences. First, we investigated C–D inferences that are *also* supported by a curated relationship in CTD, which we refer to hereafter as “curated inferences” (curated relationships were derived from one or more references independent of those involved in the inference). Second, we examined C–D inferences generated from randomized C–G interactions.

Of the C–D inferences in CTD, 3,542 are supported by a curated relationship and 334,942 are not. All but eight curated and 84 novel C–D inferences (those currently without curated support) have a Bonferroni-corrected *W_XYA_* score greater than 2.0 (-log_10_(0.05)). The distributions of Bonferroni-corrected *W_XYA_* scores among curated and novel inferences had significantly different means (p<0.001, Welch's t-test). Curated C–D inferences had a mean *W_XYA_* score of 12.46+/−17.85 (median = 6.27) and novel inferences had a mean of 5.81+/−3.76 (median = 4.88). Since the means were significantly different, we applied a simple binary classifier to determine which novel inferences were most like curated inferences by selecting those novel inferences with *W_XYA_* scores greater than the median of curated inferences. Among these, 74,119 (22.13%) of all novel inferences scored above the threshold. We also applied the same threshold to all curated inferences and found that the sensitivity was, by definition, 50%. The specificity for the classifier could not be calculated since there were no known negative C–D relationships.

Finally, we evaluated the significance of C–D inferences by comparing the distribution of *W_XYA_* scores made from CTD data and those that can be made after randomizing C–G interactions. This analysis sought to determine whether we would expect to find the observed distribution of *W_XYA_* scores by chance. We used a shuffling technique that preserves the underlying power-law distribution of node degrees [20]. Preserving the distribution of degrees was important, as we would expect to find some high degree hub nodes in any biological network. The shuffling procedure randomly selected two C–G interactions and then switched the genes with which the two chemicals interacted. Shuffling was repeated a total of 1,000,000 times before C–D inferences were made and *W_XYA_* calculated. The entire procedure was repeated three times. Since the topological properties of the networks were preserved, the distributions of the Bonferroni-corrected *W_XYA_* scores from the three shuffled networks were similar to the non-shuffled data. We compared the significance of C–D inferences made in non-shuffled network with inferences made from the three shuffled data sets. Among the 338,484 inferences that were made in the non-shuffled network, 94,222 (27.8%), 93,365 (27.6%) and 94,370 (27.9%) were also made in the first, second and third shuffled networks, respectively (Table 4). The mean differences of the *W_XYA_* score for inferences in the shuffled networks were significantly lower than those from the non-shuffled network. Among curated inferences from the non-shuffled network, the mean differences were 6.68, 6.96 and 6.86 in the three shuffled networks, respectively. Similarly for novel inferences, the mean differences were 0.80, 0.79 and 0.78, respectively. These results demonstrate that inferences from the non-shuffled network consistently scored higher than inferences from the shuffled network and therefore, may not be due to chance.

**Table 4:** Comparison of Specific Inferences in Non-Shuffled and Shuffled Networks.

Evidence For Inference	Number of Inferences in Real Network	Shuffled Network #1		Shuffled Network #2		Shuffled Network #3	
		Matching Inferences	Mean Difference W_xya_ (*p*-value)	Matching Inferences	Mean Difference W_xya_ (*p*-value)	Matching Inferences	Mean Difference W_xya_ (*p*-value)
Curated	3,542	2,000	6.68 (p<0.0001)	1,977	6.96 (p<0.0001)	1,986	6.86 (p<0.0001)
Novel	334,942	92,222	0.80 (p<0.0001)	91,338	0.79 (p<0.0001)	92,384	0.78 (p<0.0001)
TOTAL	338,484	94,222	0.92 (p<0.0001)	93,365	0.92 (p<0.0001)	94,370	0.91 (p<0.0001)

There were a total of 232,072, 232,347 and 233,515 inferences made in the first, second and third shuffled networks.

### Relevance of Chemical-Disease Inferences

One of the most valuable abilities of CTD is facilitating development of novel and potentially biologically important hypotheses about C–D relationships through transitive inferences. By virtue of CTD's unique data curation and integration, these inferences can be further validated and explored through other associated data, including pathways in which the unique underlying gene sets are involved and the functional roles of these genes.

#### Chemical-Pathway analysis

Chronic diseases have many possible etiologies that reflect genetic predisposition and varied environmental factors that perturb important biological pathways. Diverse environmental factors have been suspected in playing a role in breast cancer, although the underlying mechanisms are often not well understood. CTD contains many C–D relationships for breast cancer including two that involve the ubiquitous compounds, BPA and arsenic. To gain insight into the basis of these relationships, we computed enriched pathways from the underlying gene sets using Ingenuity Pathways Analysis (IPA; Ingenuity Systems, Inc.).

BPA is a ubiquitous endocrine disruptor that is used to manufacture polycarbonate and resin-lined food containers, polyvinyl chloride and some dental sealants [21]. Large-scale production and incorporation of BPA into products used to store food and water has resulted in human exposures. Low levels of BPA are detectable throughout the population of the United States. The consequences of developmental exposure to BPA are not well understood, although several lines of evidence suggest there is reason for concern: BPA has been detected in blood of pregnant women, breast milk of lactating women, and breast- and bottle-fed infants; BPA easily crosses the placenta; infants cannot efficiently metabolize BPA; nontoxic doses of BPA cause epigenetic modifications; and exposure has been associated with a range of endocrine related conditions such as abnormal sex-specific behavior and reproductive development, and cancers such as breast cancer [22]. The mechanisms underlying these effects remain largely unknown.

**Figure 3:**
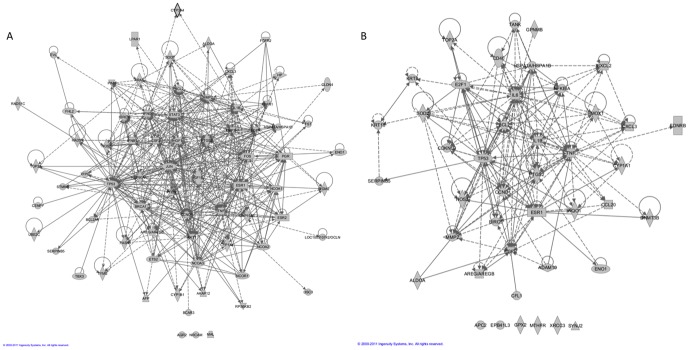
Network of gene regulatory and protein-protein interactions from Ingenuity Pathways Analysis for two chemical-disease inferences. A) Network of 73 genes used to make the curated inference between BPA and breast neoplasms, and B) network of 43 genes used to make the novel inference between arsenic and breast neoplasms.

CTD's BPA-breast neoplasm relationship is supported by direct evidence from the literature but is also inferred based on a novel set of 73 common interacting genes. Notably, the direct evidence in the literature does not include a proposed etiological mechanism. To assess the overall potential connectivity among the 73 genes, we constructed a network containing 70 of these genes based on known protein-protein and gene regulatory interactions using the IPA network explorer tool (Figure 3A). Although this network shows all known interactions, we also examined canonical pathways to provide context for subsets of interactions in the network. To identify canonical pathways that are enriched among the 73 genes, we conducted an IPA core analysis. Consistent with its known role as an endocrine disruptor, two of the three top enriched pathways among the 73 genes were involved in glucocorticoid receptor signaling (p-value = 1.21×10^−22^) and estrogen-dependent breast cancer signaling (p-value = 9.13×10^−14^). Among the 57 genes in the latter pathway, 11 have CTD-curated interactions with BPA and breast cancer (*AKT1, CCND1, CYP19A1, EGFR, ESR1, ESR2, FOS, IGF1, IGF1R, JUN* and *KRAS*; Figure 3A). In addition, another 11 genes in the estrogen-dependent breast cancer signaling pathway have curated interactions with BPA but do not currently have curated relationships to breast cancer. These data demonstrate that 38 percent of the genes (22/57) thought to be involved in estrogen-dependent breast cancer signaling are known to interact with BPA in CTD (p-value = 1.08×10^−18^).

Arsenic is a global environmental health threat. It is a known human carcinogen and a suspected endocrine disruptor. Over 500 million people are at risk of exposure to arsenic from contaminated groundwater in eastern India and Bangladesh alone [23]. It is estimated that more than 100,000 individuals in New England are exposed to drinking water levels in wells that exceed federal standards of 10 ppb [24]. Human health effects associated with arsenic exposure include lung, bladder and skin cancer [25]. Low-level exposures to arsenic (<10 ppb) have potentially adverse biologic effects such as alterations in cell cycle kinetics, cell proliferation, endocrine signaling, cell signaling, innate immune response and DNA repair processes [26], [27], [28], [29].

CTD contains many curated arsenic-disease associations including those mentioned above. However, unlike in the BPA data, the arsenic-breast neoplasm association is currently strictly inferred, based on 43 common interacting genes (Figure 3B). To assess the overall potential connectivity among these genes, we were able to construct a pathway that contained 37 of the genes. The 43 genes used to make the novel inference between arsenic and breast neoplasms were subjected to Ingenuity core analysis and yielded several enriched pathways. The most significant enriched pathway was the aryl hydrocarbon receptor (AHR) signaling pathway (p-value = 1.62×10^−12^), which contained ten of the 43 genes (*CCND1, CDKN1B, CYP1A1, E2F1, ESR1, IL6, IL1B, NQO1, TNF* and *TP53*). An additional 27 of the 154 genes in the Ingenuity AHR signaling pathway also have curated arsenic interactions (p-value = 7.78×10^−28^). Although many of these genes are not currently associated with breast cancer in CTD, many AHR ligands have curated mechanistic and potentially therapeutic relationships with breast cancer such as benzo[a]pyrene and indole-3-carbinol, respectively. Consistent with its suspected role as an endocrine disruptor, the estrogen-dependent breast cancer signaling pathway was marginally enriched (p-value = 0.043) because three genes (*EGFR, ESR1* and *CCND1*) were used to make the inference. The activated ESR1 can activate the transcription of many targets. In fact, arsenic has been demonstrated to be an endocrine disruptor [30]. Of the 57 genes in the estrogen-dependent breast cancer signaling pathway, 13 of them have characterized interactions with arsenic in CTD (p-value = 6.93×10^−10^), 11 of which are not currently associated with breast cancer in CTD.

#### Gene Ontology Enrichment Analysis

The mechanisms by which a chemical may influence disease susceptibility can be investigated by analyzing the genes used to make the C–D inference to find enriched functional annotations. An examination of the 73 genes used to make the novel inference between BPA and breast neoplasms had many enriched Gene Ontology (GO) Biological Processes pertinent to cancer such as “negative regulation of apoptosis” (Table S1A). In addition to these cancer-associated annotations, 12 genes (*BCL2, BRCA1, CCND1*, *ESR1, ESR2, FOS, IL1B, KRAS, PTEN, PTGS2, STAT3, TNF*) were enriched for “response to steroid hormone stimulus” (FDR-adjusted p-value = 7.3×10^−6^). We observed similar cancer-relevant enriched annotations for the 43 genes used to make the inference between arsenic and breast neoplasms (Table S1B). “Response to steroid hormone stimulus” was also enriched among this data set and annotated to 12 genes (*BCL2, CCND1, ESR1, HMOX1, IL1B, IL6*, *PTGS2, TNF*; FDR-adjusted p-value 1.8×10^−4^).

### CTD Web Interface

**Figure 4:**
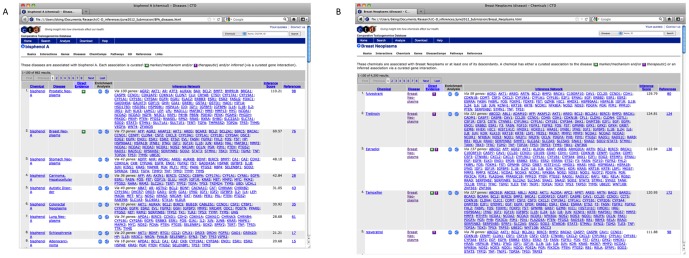
CTD web interface data tables with ranked C–D relationships. **A**) all curated and inferred C–D relationships for BPA (first page only), and **B**) all curated and inferred C–D relationships for breast neoplasms (first page only) sorted by descending values of *W_XYA_* (“Network Score”). Chemical and disease names along with gene symbols are hyperlinked to the CTD detail pages for the chemical, disease and gene, respectively. The direct evidence column is used to display a “M” and/or “T” symbols to indicate whether C–D relationship is curated and the type of the relationship. The “M” symbol indicates that the chemical correlates with disease (marker) or plays a role in the etiology of the disease (mechanism) and the “T” indicates that the chemical has a known or potential therapeutic role in the disease. The number of references in the last column is a hyperlink to the list of references that document the C–G, G–D or C–D relationship. Any references used to make a curated relationship are marked with a “M” or “T” symbol. Users may sort the tables by clicking on the column headings and may also export the tables in Excel or comma-separated, tab-separated or XML text files.

C–D inferences are represented in two ways in the CTD web interface. First, a table of all disease inferences for a given chemical is displayed under the “Disease” tab associated with that chemical (Figure 4A). The value of *W_XYA_* for an inference is displayed in the column labeled “Network Score”. Diseases are sorted by default to show curated relationships first, followed by descending network scores. Links within the table can be used to learn more about the disease, any of the genes or the available references for the disease relationship. Second, a table of all chemical inferences for a given disease is displayed under the “Chemicals” tab associated with that disease (Figure 4B).

## Discussion

We describe the first application of local-network-based statistics to C−D transitive inferences. Network-based statistics encapsulate several concepts that are important for ranking transitive inferences. First, they reward inferences with more common neighbors. For example, an inferred relationship between a chemical and disease based on a single gene has a lower score than an inference based on multiple genes. Second, these statistics take into account the number of edges (node degree) for each node (i.e., chemical, gene(s) and disease) involved in an inference so that hub nodes contribute less to the overall score. By taking into account node degree, inferences with the same number of underlying genes but greater number of hubs among those genes will have a lower score. Hub nodes are certainly expected as the CTD network was found to be a scale-free random network like other biological networks. The weighted common neighbor statistic, *W_XYA_*, takes into account the *number* of edges *and* the local topology of each node in an inference network. In contrast to the other methods evaluated, inferences ranked using the *W_XYA_* score correlated most strongly with curated disease relationships. In addition, *W_XYA_* scores for curated and novel inferences from non-shuffled networks were consistently higher than those from shuffled networks.

The common neighbor statistic we applied was designed to help users rank the many transitive disease inferences in CTD. Since these inferences are based on curated relationships from the primary literature, they are presumed to be potentially biologically important. However, the inference scores cannot be interpreted as a relative measure of correctness primarily because the underlying network information is incomplete and reflects both the bias inherent to our curation prioritization process and the bias among the chemical, gene, and disease data in the published literature. A consequence of this incompleteness is that an inference that is also supported by curated data may not always score higher than a novel inference.

That being said, CTD provides other important lines of evidence that can be evaluated in conjunction with disease inference for prioritization and hypothesis development. First, the unique gene sets that underlie chemical-disease inferences can be subjected to additional study, such as IPA to determine if associated canonical pathways and their functions are consistent with the inferred diseases. In the examples presented, gene sets underlying arsenic- and BPA-breast neoplasm inferences were significantly associated with IPA's “estrogen-dependent breast cancer signaling pathway,” consistent with the suspected endocrine-disrupting properties of both of these chemicals. Second, CTD provides enriched GO analyses for chemicals that provide insights into the biological processes and molecular functions that may be affected by a chemical via their interacting genes. In the examples presented, enriched terms such as “regulation of cell cycle,” “apoptosis” and “response to steroid hormone stimulus” were significantly and logically enriched among arsenic- and BPA-breast neoplasm inferences. Finally, CTD also integrates curated pathways from the KEGG and Reactome databases. These pathways are associated with chemicals when their constituent genes have curated interactions with a chemical. Collectively, CTD provides a unique structure for building inferences among chemical, gene and disease data that are otherwise disconnected in the literature, and couples it with additional biological information, such as biological function and molecular pathways, that can be used to strengthen hypotheses for further study.

To our knowledge, this study is the first to apply local network topology to analyze chemical, gene and disease networks. Other studies incorporated different evidence types or focused on *either* chemical-gene interactions *or* gene-disease relationships. For example, the CoPub database scores transitive inferences among drugs, genes and chemicals based on the number of citations that report an interaction [4]. However, in our experience, citation evidence does not always correlate well with the significance of a biological finding (e.g., new discoveries). The ChemProt database uses chemical-gene interactions, including those from CTD, to establish links between chemicals and diseases, but does not make novel inferences [31]. DiseaseNet integrates networks of protein-protein interactions with gene-disease relationships, but does not contain chemical data [32]. None of these resources apply statistical measures to predict novel data relationships by taking into account local network topology information.

Development of predictive models benefits from the existence of a gold standard, or validated data. Although major advances have been made in environmental health with the increasing use of computational approaches, high-throughput technologies and integrative resources, questions about how the environment affects human health in the context of diverse genetic backgrounds remain largely unanswered. Consequently, a gold-standard data set describing the etiologies of environmentally influenced diseases does not exist. Such models will evolve and improve as data sources and our understanding of them become richer. Here we describe a method to rank novel chemical-disease inferences using a unique combination of statistical approaches that consider the local topology of chemicals, genes and diseases. We show that this ranking more strongly reflects the published literature than other methods tested and that additional data sets in CTD can be used as lines of evidence for evaluating and prioritizing chemical-disease inferences for further exploration. We will continue to draw from other statistical approaches and explore the integration of additional data sources into our analyses as we seek to enhance the hypothesis-generating potential of CTD.

## Methods

### CTD Network Data

CTD (http://ctdbase.org) provides manually curated data from peer-reviewed scientific literature that describe chemical-gene interactions from vertebrates and invertebrates, chemical-disease relationships and gene-disease relationships. We used data from the July 2011 release of CTD that contained 283,236 curated interactions between 6,327 chemicals and 19,182 genes and proteins in 341 organisms. 176,999 of these interactions were unique chemical-gene pairs. In addition, there were a total of 5,251 curated gene-disease relationships between 3,564 genes and 3,338 diseases, and 6,682 curated chemical-disease relationships between 6,225 chemicals and 1,041 diseases.

### Hypergeometric Mutual Clustering Coefficient

The hypergeometric mutual clustering coefficient, *C_xy_*, for the inferred relationship between nodes *x* and *y* was calculated according to Roth and Goldberg and shown in equation 1 using notation that follows Li and Liang [12], [13]. In equation 1, *n_x_* and *n_y_* are the number of edges for nodes *x* and *y*, respectively, *m* was the number of mutual neighboring nodes, and *N* was the total number of chemicals, genes and diseases with any interaction in CTD.
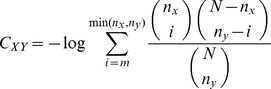
(1)


### Common Neighbor Statistics

The two common neighbor statistics, *p_1_* and *p_2_*, were calculated for inferred relationships between nodes *x* and *y* according to Li and Liang as shown in equations 2 and 3 [13]. In equation 2, *n_x_* and *n_y_* are the number of edges for nodes *x* and *y*, respectively, *m* was the number of mutual neighboring nodes, and *N* was the total number of chemicals, genes and diseases with any interaction in CTD. In equation 3, *A* was the set of genes that connect the chemical and disease and *n_i_* are the number of edges for the particular connecting gene. All values for *p_1_* and *p_2_* are –log_10_ transformed. The *p_1_* and *p_2_* probability distributions were combined into an aggregate statistic using the logarithmic opinion pool approach shown in equation 4 [33]. As shown in equation 5, *S_XYA_* is a log_10_-transformed form of *p(θ)* with *w_1_* =  *w_2_* = ½ and *k* = 1. *W_XYA_* is also a log_10_-transformed form of *p(θ)* with *k* = 1, but *w_1_* and *w_2_* are a function of m as shown in equation 6.
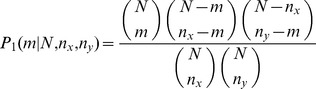
(2)

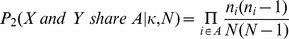
(3)

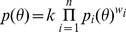
(4)

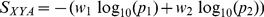
(5)

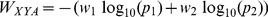
(6)where
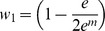
and
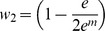



*C_xy_*, *S_XYA_* and *W_XYA_* were calculated using a Java program that used the Apache Commons Mathematics library (http://commons.apache.org/math) to compute large factorials using the Gamma function.

*C_xy_*, *S_XYA_* and *W_XYA_* were calculated using a Java program that used the Apache Commons Mathematics library (http://commons.apache.org/math) to compute large factorials using the Gamma function.

### Gene Ontology Enrichment Analysis

Enriched Gene Ontology terms annotated to genes used to make the arsenic and BPA breast neoplasms C–D inferences were identified using DAVID (http://david.abcc.ncifcrf.gov) [34].

## Supporting Information

Figure S1 **Degree distribution for the CTD network.** The slope of a line fit to these data was -0.80 (*r^2^* = 0.94).
(TIFF)

Figure S2 **Cumulative frequency distributions of ties by method** ***C_xy_*** **,** ***p_1_*** **,** ***p_2_*** **,** ***S_XYA_*** **and** ***W_XYA_*** **.** In all, Cxy had 127,311 ties among 3,866 chemicals; *p_1_* had 127,309 ties among 3,866 chemicals; *p_2_* had 192,923 ties among 5,395 chemicals; *S_XYA_* had 41,767 ties among 3,583 chemicals; and *W_XYA_* 41,768 ties among 3,583 chemicals.
(TIFF)

Figure S3 **Cumulative number of curated inferences according to rank order among top 100 scoring C-D inferences by** ***S_XYA_*** **(blue) and** ***W_XYA_*** **(red).** The plot shows that curated inferences have a higher rank order when scored by *W_XYA_* than *S_XYA_*.
(TIFF)

Table S1 Lists of enriched Gene Ontology Biological Process FAT terms with a FDR-adjusted p-value<0.05 for two C–D inferences. **A**) Enriched terms for 73 genes used to make the curated inference between BPA and breast neoplasms, and **B**) enriched terms for 43 genes used to make the novel inference between arsenic and breast neoplasms.
(XLSX)
