# Supplementary figures and images for: Ranking Transitive Chemical-Disease Inferences Using Local Network Topology in the Comparative Toxicogenomics Database
Source: PLoS One. 2012 Nov 7;7(11):e46524. doi: 10.1371/journal.pone.0046524 (PMC3492369; doi:10.1371/journal.pone.0046524)

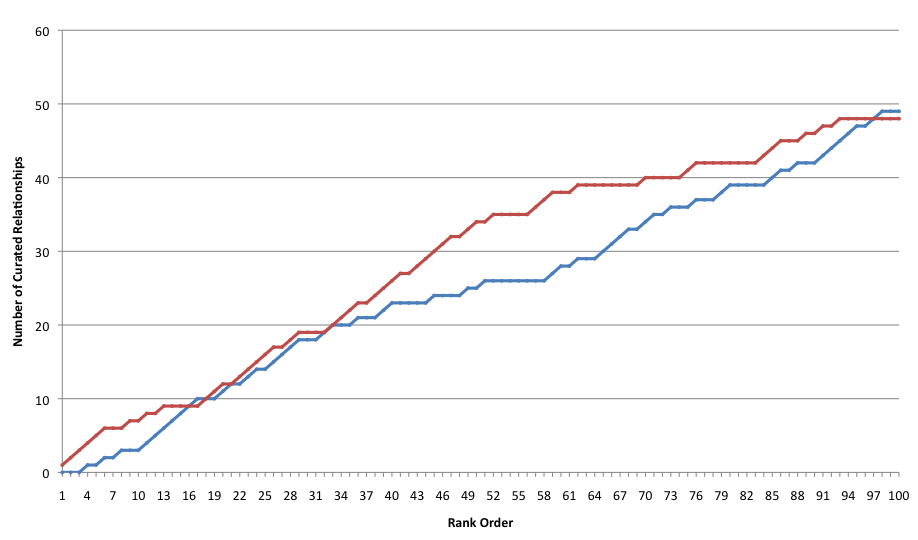

Supplement: Figure S3 — Cumulative number of curated inferences according to rank order among top 100 scoring C-D inferences by SXYA (blue) and WXYA (red). The plot shows that curated inferences have a higher rank order when scored by WXYA than SXYA. (TIFF) [file pone.0046524.s003.tiff]
